# Transrectus sheath pre-peritoneal (TREPP) procedure versus totally extraperitoneal (TEP) procedure and Lichtenstein technique: a propensity-score-matched analysis in Dutch high-volume regional hospitals

**DOI:** 10.1007/s10029-020-02291-7

**Published:** 2020-10-16

**Authors:** T. L. R. Zwols, N. Slagter, N. J. G. M. Veeger, M. J. W. Möllers, D. A. Hess, E. Jutte, H. T. Brandsma, P. H. J. M. Veldman, G. G. Koning, H. H. Eker, J. P. E. N. Pierie

**Affiliations:** 1 Department of Surgery, Medisch Centrum Leeuwarden, Leeuwarden, The Netherlands; 2 Department of Clinical Epidemiology, Medisch Centrum Leeuwarden, Leeuwarden, The Netherlands; 3 Department of Surgery, Nij Smellinghe, Drachten, The Netherlands; 4 Department of Surgery, Antonius Ziekenhuis, Sneek, The Netherlands; 5 Department of Surgery, Tjongerschans Heerenveen, Heerenveen, The Netherlands; 6 Department of Surgery, Ikazia Ziekenhuis, Rotterdam, The Netherlands; 7 Postgraduate School of Medicine, University Medical Centre Groningen, Groningen, The Netherlands; 8 The University Medical Centre Groningen, University of Groningen, Groningen, The Netherlands

**Keywords:** TEP, Lichtenstein, TREPP, Open preperitoneal, Inguinal hernia repair, Groin hernia

## Abstract

**Purpose:**

Results of the most commonly used inguinal hernia repair techniques often originate from expert centers or from randomized controlled studies. In this study, we portray daily-practice results of a high-volume, regional surgical group in the Netherlands, comparing TREPP (open (posterior) transrectus sheath pre-peritoneal) with Lichtenstein (open anterior) and TEP (endoscopic (posterior) totally extraperitoneal). We hypothesize that the TREPP shows more favorable outcome compared to the current gold standard procedures: TEP and Lichtenstein.

**Methods:**

Between January 2016 and December 2018, 3285 consecutive patients underwent surgical treatment and were included for analysis. The outcome measures were postoperative pain, recurrence rate and other surgical complications. Propensity-score matching was used to address potential selection bias.

**Results:**

After propensity-score matching, there was no statistically significant difference in postoperative pain in the TREPP group compared to the Lichtenstein group (TREPP 7.3% versus Lichtenstein 6.3%; *p* = 0.67) nor in TREPP compared to TEP (TREPP 7.4% versus TEP 4.1%; *p* = 0.064). There was no statistically significant difference in recurrences in the TREPP group compared to Lichtenstein (3.8% vs 2.5%; *p* = 0.42), nor in the TREPP versus TEP comparison (3.9% vs 2.8%; *p* = 0.55)

**Conclusion:**

This study compares TREPP with Lichtenstein and TEP in the presence of postoperative pain, recurrences and other adverse outcomes. After propensity-score matching, no statistically significant difference in postoperative pain or recurrences remained between either TREPP compared to Lichtenstein, or TREPP compared to TEP. Based on these results, TREPP, Lichtenstein and TEP showed comparable results in postoperative pain, recurrences and other surgical site complications.

## Introduction

Inguinal hernia repair is one of the most frequently performed surgical procedures worldwide with up to 20 million patients annually [1]. In the Netherlands, approximately 30,000 patients undergo inguinal hernia repair each year [2]. The current recommendation for patients who experience symptoms of inguinal hernia such as pain or mechanical complaints is elective inguinal hernia repair. International guidelines recommend conservative treatment without surgery if the patient has no complaints. Studies have shown, however, that approximately 70% of patients will have an increase in complaints and will eventually require inguinal hernia repair [3, 4].

In the current guidelines, the preferred surgical technique in any elective inguinal hernia repair depends on many different characteristics and can differ between patients. In general, a surgical technique that includes mesh to strengthen the abdominal wall is recommended [1]. The totally extraperitoneal (TEP) procedure is the recommended endoscopic technique for elective inguinal hernia repair. The Lichtenstein technique is recommended for patients who are not fit for general anesthesia, in case of recurrence or when minimal invasive equipment or experience is lacking. In patients where mesh placement is impossible or where circumstances make mesh placement less opportune, the Shouldice technique is the recommended non-mesh technique for inguinal hernia repair [1].

TEP and Lichtenstein show similar perioperative complications and operating times [5]. After the introduction of mesh placement in elective inguinal hernia repair, the rate of recurrences dropped. With this lower rate of recurrences, another outcome of inguinal hernia repair became most prevalent: chronic postoperative inguinal pain (CPIP), which is defined as any inguinal pain > 3 months after surgery [6]. The overall incidence of CPIP is approximately 11% [7]. The prevalence of patients that are affected with CPIP in daily activities or work ranges from 0.5 to 6% [1]. The TEP shows slightly better results in the development of chronic postoperative inguinal pain (CPIP) and return to normal daily activities in comparison to the Lichtenstein technique [5, 8].

For the Lichtenstein technique, prevalence varies up to 29% [5, 8–10]. After TEP, these percentages vary up to 12% [5, 8, 9]. For the transrectus sheath pre-peritoneal (TREPP) procedure, there is less literature available, but the existing literature suggests a lower percentage of pain around 5% [11].

Most results originate from high-volume expert centers where one specific technique is used or from randomized studies that usually include a specific subset of the general population. The cumulative results of these specific studies have become the textbook-outcome results but may differ from the general daily practice. In this study, we portray daily-practice results of inguinal hernia surgery performed in four high-volume regional hospitals in the Netherlands, both teaching and non-teaching, on TREPP, TEP and Lichtenstein. The aim of this study is to evaluate textbook-outcome results on inguinal hernia surgery in the daily practice in regional hospitals, to provide both doctors and patients with more insights on the risk of complications after inguinal hernia surgery. We hypothesize that TREPP results in a more favorable outcome (postoperative pain, recurrence) compared to the gold standard procedures TEP and Lichtenstein.

## Patients and methods

### Study design

The Medical Ethics Committee of our institution (RTPO Leeuwarden, The Netherlands) confirmed that this retrospective study could be carried out without the need for ethical review, and the institutional boards of the four hospitals approved the execution of the study without the need for consent in accordance with Dutch regulations. All participating centers provided data of experienced hernia surgeons (at least 100 inguinal hernia procedures performed) performing or supervising the surgical treatment. The aim of this study was to evaluate the daily practice in inguinal hernia surgery in four teaching and non-teaching hospitals in the northern region of the Netherlands. The primary outcome of the study was to evaluate the development of postoperative pain. The secondary outcomes are surgical site occurrences (recurrence, haematoma, seroma, bleeding, wound infection, abscess, urinary tract infection, ileus).

### Retrospective database

All patients with a primary or recurrent, direct or indirect, inguinal or femoral hernia, in an acute or elective setting were included in the database. Patients who did not undergo surgical treatment were excluded from the database. Patients were operated with different anterior or posterior techniques, e.g., Lichtenstein, TEP, TAPP, TREPP, TIPP, Bassini, Shouldice, Stoppa, Rutkow-Robbins, Fabricius. The choice of technique was dependent on the surgeon and on whether or not it concerned a primary or recurrent hernia. For this study, analyses were performed on all adult male patients with primary, unilateral inguinal hernia, who were treated in the elective setting.

### Outcome measures

Patients were scheduled for regular follow-ups at the outpatient clinic at two–six weeks postoperatively. More visits were scheduled only in case of adverse events. Every outcome that was mentioned in the electronic patient file was noted in the database. For postoperative pain specifically, patients scored a “yes” if they: visited the outpatient clinic after a regular follow-up because of inguinal pain; received pain treatment or had any further pain evaluation (e.g., ultrasonography, MR-imaging, referral to pain specialist).

### Statistical analysis

To address potential selection bias due to the observational nonrandomized study design, we implemented propensity-score matching to achieve a more balanced study cohort. In this, patients treated with TREPP were 1:1 matched to patients treated with Lichtenstein, using their probability to receive TREPP, i.e., the propensity score for TREPP. Matching was performed with a 0.01 maximum allowed difference in the exact propensity scores in a ‘pair of patients’ treated with TREPP and Lichtenstein. Propensity score of individual patients was estimated using multivariable logistic regression with covariates describing condition at baseline. In addition, patients treated with TREPP were matched to TEP used the same procedure.

Subsequent analyses were performed using the matched cohorts. Percentages of outcome were calculated and exact odds ratios with 95% confidence intervals estimated. For this, exact conditional logistic regression was used to control for potential lack of independence due to matched pairs.

Baseline characteristics between groups were analyzed using Chi-square test and a two-tailed Fisher exact test. Outcome measures in both cohorts were analyzed using a two-tailed Fisher exact test. *p* values less than 0.05 were considered statistically significant. Statistical analysis was performed with SAS 9.2 (SAS Institute Inc. Cary, NY, USA).

## Results

### Patient characteristics

**Fig. 1:**
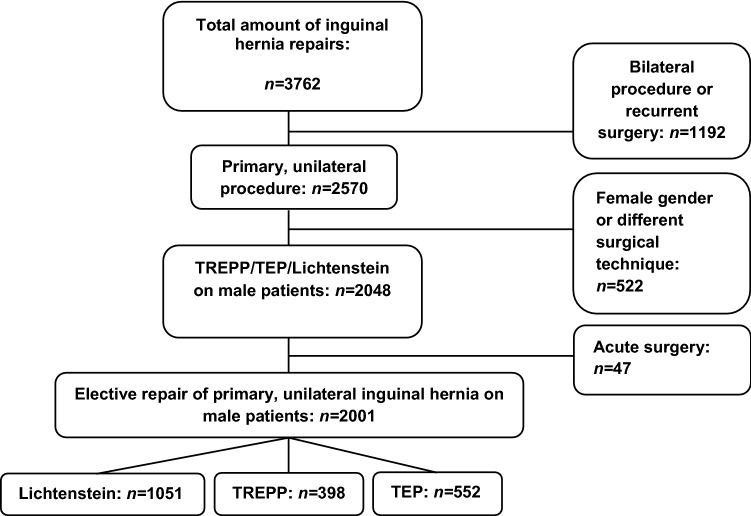
Flowchart of total inguinal hernia repairs within the selected timeframe

In the period between January 1st, 2016 and December 31st, 2018, a total of 3762 inguinal hernia repairs were performed. Surgeries were performed in four regional hospitals, one teaching and three non-teaching. The flowchart of selected patients for analysis in this study is shown in Fig. 1.

### Baseline characteristics

Before propensity-score matching (PSM), the cohort undergoing the TREPP procedure (*n* = 398) was younger (mean age 57.0; 18–88 versus 63.7 years; 18–95) than the Lichtenstein cohort (*n* = 1051). The TEP cohort (*n* = 552) had the lowest mean age (54.3 years; 18–88). Baseline patient characteristics are shown in Table 1.

**Table 1:** Patient characteristics

	TREPP (*n* = 398)	TEP (*n* = 552)	Lichtenstein (*n* = 1051)
Mean age in years (SD)	57 (14.6)	54 (15.4)	64 (15.1)
ASA class (%)			
I (%)	163 (41)	215 (39)	286 (28)
II (%)	201 (51)	295 (54)	519 (51)
III (%)	34 (8)	39 (7)	208 (20)
IV (%)	0 (0)	2 (0)	14 (1)

### TREPP versus Lichtenstein

The presence of postoperative pain in the Lichtenstein cohort (before PSM) was significantly lower than in the TREPP cohort (Lichtenstein 4.2% versus TREPP 7.3%; *p* = 0.022). There was no significant difference in the number of recurrences in both cohorts (Lichtenstein 2.6% versus TREPP 3.8%; *p* = 0.22) The cumulative number of other adverse outcomes (e.g., hematoma, seroma, infection, urinary tract infection) was significantly higher in the Lichtenstein cohort (13.0%) as compared to the TREPP cohort (9.1%; *p* = 0.037). These numbers are shown in Table 2.

**Table 2:** Postoperative outcomes TREPP versus Lichtenstein before propensity-score matching

	TREPP (*n* = 398)	Lichtenstein (*n* = 1051)	*p* value
Postoperative pain, *n* (%)	29 (7.3)	44 (4.2)	0.022
Recurrence, *n* (%)	15 (3.8)	27 (2.6)	0.22
Surgical Site Occurrence, *n* (%)	36 (9.1)	137 (13.0)	0.037

When comparing TREPP with Lichtenstein after PSM, all patients undergoing the TREPP procedure (*n* = 398) could be paired with a patient undergoing the Lichtenstein procedure, which created two groups with a total of 796 patients. The mean age of the non-matched group was significantly higher than the mean age in the matched pair group (mean age 68 versus 57; *p* < 0.001). The ASA-classification was significantly higher in the non-matched group (*p* < 0.001). When comparing TREPP versus Lichtenstein after PSM, the patients undergoing the TREPP technique did not experience significantly more postoperative pain as compared to the Lichtenstein group (TREPP 7.3% versus Lichtenstein 6.3%; *p* = 0.67). The percentage of recurrences in the TREPP cohort was 3.8%, which did not differ significantly compared to the Lichtenstein cohort (2.5%; *p* = 0.42). The total number of adverse outcomes did not significantly differ between the two groups (TREPP 9.1% versus Lichtenstein 12.1%; *p* = 0.20). The odds ratio after exact conditional logistical regression for postoperative pain was 1.2 (confidence interval (CI) 0.65–2.14; *p* = 0.67), for recurrence 1.5 (CI 0.63–3.73; *p* = 0.4), for combined number of other adverse outcomes 0.7 (CI 0.44–1.18; *p* = 0.2). These results are shown in Table 3.

**Table 3:** Comparison in postoperative outcomes in Lichtenstein and TREPP after propensity-score-matched analysis

	TREPP (*n* = 398)	Lichtenstein (*n* = 398)	*p* value
Postoperative pain, *n* (%)	29 (7.3)	25 (6.3)	0.67
Recurrence, *n* (%)	15 (3.8)	10 (2.5)	0.42
Surgical site occurrence, *n* (%)	36 (9.1)	48 (12.1)	0.20

### TEP versus TREPP

Prevalence of postoperative pain in the TEP cohort did not differ significantly compared to the TREPP procedure (TEP 5.1% versus TREPP 7.3%, *p* = 0.17) and the prevalence of recurrent inguinal hernia in the TEP cohort was 2.5% (no significant difference with the TREPP cohort, 3.8%; *p* = 0.34). The cumulative total number of other adverse outcomes did not differ significantly between the two groups (TEP 10.7% versus TREPP 9.1%; *p* = 0.44). The results of TREPP versus TEP before PSM are shown in Table 4.

**Table 4:** Postoperative outcomes TREPP versus TEP before propensity-score matching

	TREPP (*n* = 398)	TEP (*n* = 552)	*p* value
Postoperative pain, *n* (%)	29 (7.3)	28 (5.1)	0.17
Recurrence, *n* (%)	15 (3.8)	14 (2.5)	0.34
Surgical site occurrence, *n* (%)	36 (9.1)	59 (10.7)	0.44

When comparing TREPP to the TEP technique after propensity-score matching, 390 pairs could be matched on propensity scores. This created two groups of a total of 780 patients. The non-matched group that remained (162 TEP patients, 8 TREPP patients) was significantly younger than the matched group (51 years versus 57 years; *p* < 0.001). Furthermore, there was no difference in ASA-classification between both groups (*p* = 0.29). When comparing the TREPP to the TEP after PSM, no significant difference was present (TREPP 7.4% versus TEP 4.1%; *p* = 0.064) in the presence of postoperative pain. Recurrences occurred in 3.9% of TREPP procedures versus 2.8% recurrences after the TEP procedure (*p* = 0.55). The cumulative total number of other adverse outcomes did not differ significantly (TREPP 9.0% versus TEP 10%; *p* = 0.71). The odds ratio after exact conditional logistical regression for postoperative pain was 1.9 (CI 0.96–3.76; *p* = 0.07), for recurrence 1.4 (CI 0.58–3.52; *p* = 0.5), for combined number of other adverse outcomes 0.9 (CI 0.51–1.49; *p* = 0.7). The results of TREPP versus TEP after propensity score matching are shown in Table 5.

**Table 5:** Comparison in postoperative outcomes in TEP and TREPP after propensity-score-matched analysis

	TREPP (*n* = 390)	TEP (*n* = 390)	*p* value
Postoperative pain, *n* (%)	29 (7.4)	16 (4.1)	0.064
Recurrence, *n* (%)	15 (3.9)	11 (2.8)	0.55
Surgical site occurrence, *n* (%)	35 (9.0)	39 (10)	0.71

## Discussion

This study presents the data of inguinal hernia repairs in four Dutch regional teaching and non-teaching hospitals from the January 1st, 2016 until December 31st, 2018. Over 3000 patients were treated for inguinal hernia in this timeframe. The aim of this study was to compare the transrectus sheath pre-peritoneal procedure (TREPP) with the two most performed elective unilateral inguinal hernia repairs: Lichtenstein and TEP, recommended by international guidelines on inguinal hernia. We performed a propensity-score-matched (PSM) analysis to lower the potential treatment selection bias known to exist with retrospective cohorts, comparing both TREPP with Lichtenstein as well as TREPP with TEP. After PSM, there was no statistically significant difference in the presence of postoperative pain when comparing TREPP with Lichtenstein or TEP. The odds ratios for postoperative pain of 1.2 (Lichtenstein) and 1.9 (TEP) seem to show a tendency in favor of TEP, but there was no statistically significant difference. There was also no statistically significant difference for recurrences or cumulative other outcome measures.

When considering the overall percentages of postoperative pain for these three procedures, there is a remarkably low prevalence of postoperative pain especially in all patients treated with the Lichtenstein procedure (4.2%) (after matching 6.3%). In the available literature, this number varies from 21.7 to 29% [5, 8, 10]. In our study, the overall prevalence of postoperative pain following TEP was 5.1% and 7.3% after TREPP (4.1% and 7.3% after matching, respectively). In the available literature, pain percentages for the TEP technique vary between 9.8 and 25% [5, 8, 10, 11] and for TREPP 5.3% is reported [11]. This difference in prevalence of postoperative pain can partly be explained by the retrospective character of this study. On the other hand, this lower percentage of postoperative pain could potentially bring nuance to the clinical significance of pain reported in clinical studies.

We investigated the differences in groups before and after propensity-score matching. It appeared that the unmatched patients in the TREPP–Lichtenstein comparison were all Lichtenstein patients, mostly of older age and higher ASA classification. It seems that this group of patients experience less pain than younger patients [12]. A possible explanation of this phenomenon could be the higher level of daily activities in younger patients and therefore a higher demand of postsurgical tissue. These daily activities, however, were not measured or noted in this study. Another possible explanation can be sought in the decrease in elasticity of tissue in older patients, possibly leading to less tension in the surgical area.

A possible explanation for the relatively low percentage of postoperative pain in the Lichtenstein population in the regional (teaching and non-teaching) hospitals included in this study is the high level of experience with this technique.

The results of this study should be interpreted cautiously. The retrospective nature of this study prevents certainty of follow-up of every patient. The authors attempted to register the number of patients with pain as accurately as possible, by purposely not using the definition of chronic postoperative inguinal pain (CPIP: pain > 3 months after surgery) [6]. Rather, “a deviation of normal follow-up” was used as a guide to report a ‘postoperative pain’. This, however, does not eliminate the possibility that patients reported their postoperative inguinal pain to the general practitioner, or in a different hospital.

The theoretical low percentages of pain following the TREPP procedure, as seen in previous cohorts [11, 13] was not seen in this study. This relatively high percentage could be explained partly by a theoretical learning curve effect. A number of surgeons implemented the TREPP procedure in this period. Although these early TREPPs are always supervised by a dedicated TREPP surgeon, a learning curve effect cannot be excluded as potential confounder.

Considering the results as well as the inherent potential biases, this study does not show significant differences between both TEP/Lichtenstein (gold standard procedures) and the TREPP procedure. The authors recommend a well designed randomized controlled clinical trial comparing the TREPP to either the TEP or the Lichtenstein to confirm the results from this study. Should the results be confirmed, however, the TREPP could play an important role in the decision-making for inguinal hernia repair. As an example, TREPP could play a useful part in recurrent hernia repair after previous anterior approach. Until now, the standard concept for treatment of recurrence after anterior approach is a posterior approach, mostly leading to a TEP (or TAPP) procedure. If the results from this study are indeed an accurate representation of the outcome, the TREPP procedure could take part in this flow chart, with possible advantages such as: single-incision access viable for intra-peritoneal inspection, pre-peritoneal location of mesh, possibility of regional anesthesia and potential lower costs due to the fact that laparoscopic equipment is not needed.

## Data Availability

Is available on request.
